# Targeted micelles with chemotherapeutics and gene drugs to inhibit the G1/S and G2/M mitotic cycle of prostate cancer

**DOI:** 10.1186/s12951-020-00756-6

**Published:** 2021-01-09

**Authors:** Yiran Zhang, Yanming Wang, Li Meng, Qingqing Huang, Yueqi Zhu, Wenguo Cui, Yingsheng Cheng, Ranlu Liu

**Affiliations:** 1 Tianjin Institute of Urology & Department of Urology, The Second Hospital of Tianjin Medical University, 23 Pingjiang Road, Hexi District, Tianjin, 300211 People’s Republic of China; 2 Department of Interventional Radiology, Shanghai Jiao Tong University Affiliated Sixth People’s Hospital, No. 600, Yishan Road, Shanghai, 200233 People’s Republic of China; 3 Shanghai Key Laboratory for Prevention and Treatment of Bone and Joint Diseases, Shanghai Institute of Traumatology and Orthopaedics, Ruijin Hospital, Shanghai Jiao Tong University School of Medicine, 197 Ruijin 2nd Road, Shanghai, 200025 People’s Republic of China; 4 Tianjin Key Laboratory of Molecular Drug Research, College of Pharmacy, Nankai University College of Pharmacy, Nankai University, Haihe Education Park, 38 Tongyan Road, Tianjin, 300353 People’s Republic of China

**Keywords:** Micelles, Targeted ligand, EPR, Prostate cancer, Gene therapy

## Abstract

**Background:**

Chemotherapy and gene therapy are used in clinical practice for the treatment of castration-resistant prostate cancer. However, the poor efficiency of drug delivery and serious systemic side effects remain an obstacle to wider application of these drugs. Herein, we report newly designed PEO-PCL micelles that were self-assembled and modified by spermine ligand, DCL ligand and TAT peptide to carry docetaxel and anti-nucleostemin siRNA.

**Results:**

The particle size of the micelles was 42 nm, the zeta potential increased from − 12.8 to 15 mV after grafting with spermine, and the optimal N/P ratio was 25:1. Cellular MTT experiments suggested that introduction of the DCL ligand resulted in high toxicity toward PSMA-positive cells and that the TAT peptide enhanced the effect. The expression of nucleostemin was significantly suppressed in vitro and in vivo, and the tumour-inhibition experiment showed that the dual-drug delivery system suppressed CRPC tumour proliferation.

**Conclusions:**

This targeted drug delivery system inhibited the G1/S and G2/M mitotic cycle via synergistic interaction of chemotherapeutics and gene drugs.
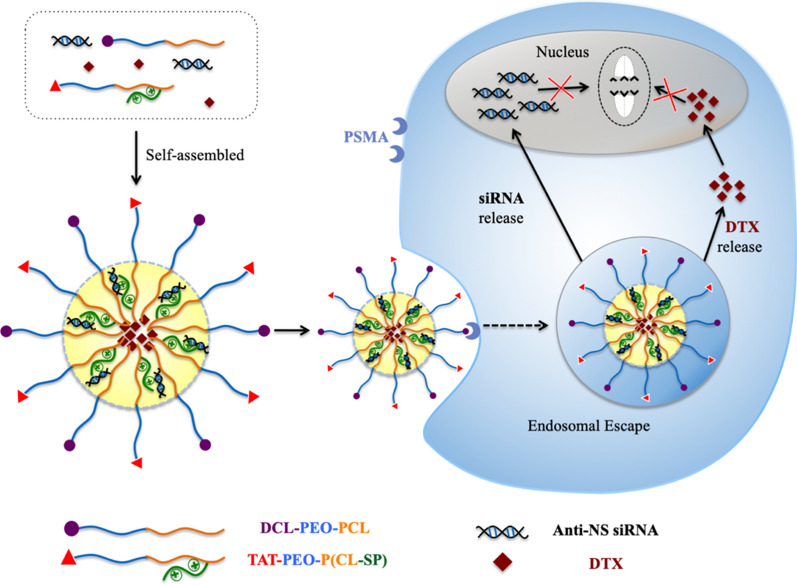

## Background

Prostate cancer (PCa) is a disease that has been reported worldwide and has become the most frequently diagnosed cancer. In 2019, newly diagnosed PCa cases were estimated at 174,650 (20% of male cancers) with 31,620 (10% of male cancers) deaths. The incidence rates of PCa remained the highest of all male cancers from 1975 to 2015 [1]. The current treatment strategy includes surgical treatment, endocrine therapy, immunotherapy, gene therapy and chemotherapy [2]. However, a large number of patients had progressed to the middle and late stages when they were first diagnosed and were no longer suitable for surgical excision. An 18-month endocrine therapy often causes the disease to progress to castration-resistant prostate cancer (CRPC), and androgen deprivation therapy (ADT) generally loses its efficacy. Immunotherapy is generally expensive or lacks universality; thus, chemotherapy and gene therapy have become the ultimate treatments for such patients.

Docetaxel (DTX), the most commonly used chemical drug, inhibits the assembly of microtubules and results in cellular mitosis cessation at the G2/M phase. The effective patient survival time was increased by 10–13 months, and the 5-year survival rate increased 8% (55% vs. 63%) [3]. Even for hormone-sensitive PCa, six cycles of DTX at the beginning of ADT also significantly prolonged the overall survival versus ADT alone. However, due to restrictions of the dosage form, this treatment did not have a high drug effect and low side effects. Many patients developed febrile neutropenia, leukopenia, thrombopenia, hypersensitivity reactions, asthenia, fluid retention, fever, fatigue, cardiac events, neuropathy, pain, and nail toxicity after receiving DTX [4, 5].

To solve the problem of the efficient application of chemical drugs, nanoparticle drug delivery systems (NDDSs) have been deeply investigated [6]. An NDDS consists of microemulsions, liposomes, nanoparticles, and micelles that effectively avoid liver- and kidney-mediated clearance during circulation via the enhanced permeability and retention (EPR) effect. Of all types of NDDS, polymer micelles are amphiphilic and self-assemble into a nanoscale spherical structure in aqueous solution. These micelles encapsulate hydrophobic drugs in their hydrophobic core. Therefore, micelles have become a good choice for enhancing the solubility of DTX in the blood. Among nanoparticles, polyethylene oxide (PEO) and polycaprolactone (PCL) are two common low-toxicity polymer biomaterials that are hydrophilic and hydrophobic, respectively [7, 8]. Polymerization on acetal-PEO using the ε-caprolactone monomer forms a long chain structure, acetal-PEO-*b*-PCL (A-PEO-PCL) [9], and DTX can be entrapped in the core after micelle self-assembly formation.

Each segment of A-PEO-PCL can be chemically modified, and the customization of chemical groups greatly expands the applications for micelles. Modification of the polymers alters the properties of the micelles, which offers the opportunity to introduce other types of therapy to achieve synergistic effects from different medications. Gene therapy is an option for suppressing tumour tolerance to chemotherapeutic drugs. Nucleostemin (NS) is a previously studied locus that is highly expressed in PCa cell lines and tissues. This P53-binding protein is located in the nucleus of stem cells and cancer cells, and the proliferation of PCa cells is significantly reduced after the silencing of NS gene expression. This inhibitory effect occurs because NS is an important G1/S checkpoint regulator, and regulation in cell mitosis is achieved via a P53-independent pathway, according to our previous research [10, 11]. NS also plays a role in PCa cells that do not express P53. Huang showed that P53 loss did not reverse the damage caused by NS loss or the ultimate fate of NS-deficient cells, but it orchestrated how the cells responded to the G2/M arrest. Cancer cells appeared to manage the NS loss condition more independently of their P53 status [12]. The introduction of anti-NS siRNA blocked the expression of the NS gene and inhibited the growth of tumour cells. A dual-drug strategy containing DTX and anti-NS siRNA inhibited the G1/S and G2/M phases of mitosis, which may achieve stronger antitumour effects.

To carry siRNA, a positively charged chemical group must be introduced. Spermine (SP) is a polyamine that contains two amino groups and two imino groups and is produced via the enzymatic catalysis of butanediamine and S-adenosylmethionine in living organisms [13, 14]. SP is widely found in most animal cells, and it is an important substance that promotes cell proliferation. SP exhibits polycationic polyamine properties, and it binds to DNA or RNA [15]. Self-assembled micelles can carry genetic drugs after the covalent linking of SP to the hydrophobic segments of the polymer [16].

As the PEO-PCL polymer has an acetal group, small molecules can be attached to the head of the hydrophilic segment of the micelles. N-[N-[(S)-1,3-dicarboxypropyl]carbamoyl]-(S)-lysine (DCL) is a ligand that can be chemically attached to one end of a polymer [17, 18]. DCL is a ligand for prostate-specific membrane antigen (PSMA), which is a transmembrane glycoprotein that is overexpressed by 100–1000 times in PCa cells and tissues and even further in metastatic and CRPC [19–21]. PSMA, also known as folate hydrolase I, is used for the sensitive diagnosis and treatment of PCa, especially CRPC. The DCL ligand was widely used as a targeting group to guide the nuclear-active particle Lu^177^ to PCa tissue in recent studies. Many research studies confirmed the clinical effects of this group [22].

Micelles precisely target PCa cells via the covalent linkage of DCL. The TAT protein, which is derived from the HIV virus and belongs to the core protein transduction domain, may also be linked to the micelles. The TAT peptide increased the transmembrane efficiency by approximately 200 times via electrical changes, and it enhanced the effect of micelle entry to the cells [23].

The present study designed a multi-functional micelle carrier. PEO-PCL was used as a framework to form micelles that carry DTX. The SP group was modified to make it positively charged to carry the gene drug anti-NS siRNA. TAT and DCL groups were used to target CRPC. The EPR effect of the micelles and the synergistic effect of the dual drugs accurately reacted on the mitotic process of tumour cells and enhanced the effective treatment of CRPC.

**Scheme 1:**
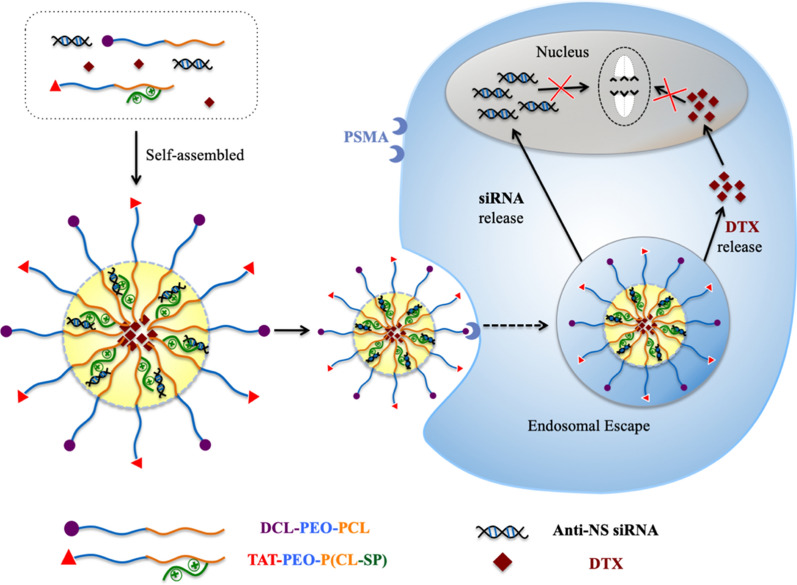
Schematic processes of co-delivery micelles that were self-assembled and modified by spermine ligand, DCL ligand and TAT peptide to carry docetaxel and anti-nucleostemin siRNA

The present study designed a synthetic acetal-PEO-*b*-PCL polymer and modified it with different ligands to carry drugs for the treatment of prostate cancer. SP was modified to transform its electrical property to positive and make it available to carry anti-NS siRNA. The DCL ligand was used to target the PCa cells, and the TAT ligand assisted the passing of polymers through the membrane of PCa cells (Scheme 1). The target micelles self-assembled and entrapped DTX and anti-NS siRNA to accurately affect the mitotic process of tumour cells and enhance the effective treatment of CRPC via the EPR effect of the micelles and the synergistic effect of dual drugs. The combination of precise therapy and gene therapy may compensate for the shortcomings of traditional chemical therapy and achieve better therapeutic effects.

## Results

### Characterization of the PEO-PCL polymers

**Fig. 1:**
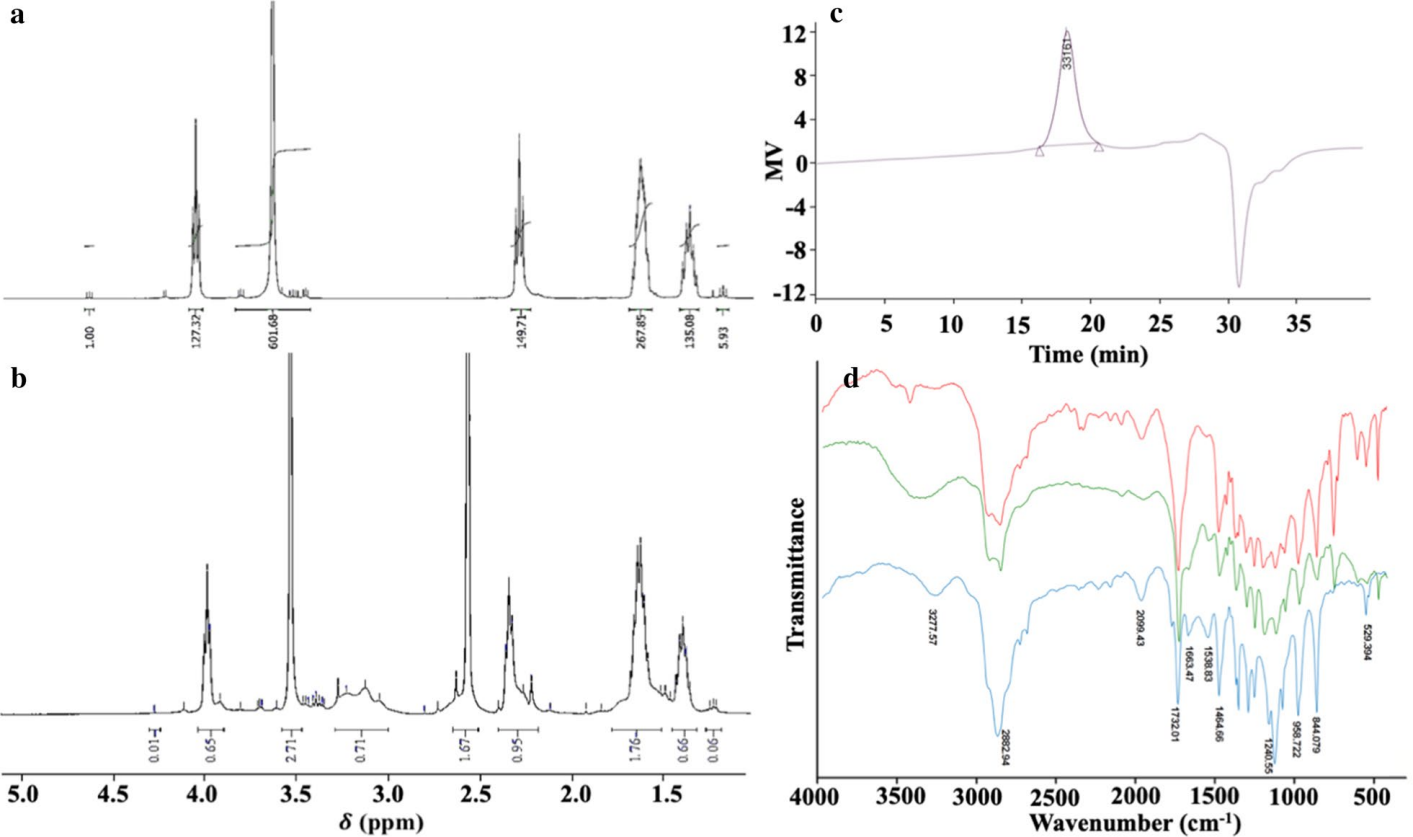
Characteristics of PEO-PCL polymers. ^1^H NMR of **a** PEO-PCL and **b** PEO-P(CL-SP). **c** GPC spectra of A-PEO-PCL. **d** FT-IR spectra of DCL-PEO-PCL (red line), TAT-PEO-PCL (green line), and A-PEO-P(CL-SP) (blue line)

The ^1^H NMR spectra and peak distribution of A-PEO-PCL are shown in Fig. 1a. The signals at 1.20 ppm were attributed to -OCH_2_CH_3_, and the signals at 4.64 ppm were attributed to a hydrogen atom on an acetal tertiary carbon. Based on the peak area of these two sets of hydrogens, the molecular weight of the polymers was calculated as 23,311 g/mol. According to the molecular weight calculated by the nuclear magnetic field, the ratio of PEO:PCL was 2.03:1. The spectra and peak distribution of PEO-P(CL-SP) are presented in Fig. 1b. The set of feature peaks of spermine at 3.0-3.3 ppm proved the successful SP modification. The gel permeation chromatography (GPC) chromatograms showed a sharp peak at 16 min with a PDI of 1.12, which indicated a molecular weight of 33,161 g/mol and a uniform product. In the FT-IR spectrum, peaks at 1730 cm^− 1^ and 2800–3000 cm^− 1^ provided evidence of the PEO-PCL chain. DCL-PEO-PCL, TAT-PEO-PCL, and A-PEO-P(CL-SP) were classified to the polyester skeleton and were indicated in the IR spectra at 1500–1560 cm^− 1^ and 1630–1690 cm^− 1^ in the presence of amide characteristic peaks, which confirmed the modifications of DCL, TAT and the arginine group.

### Characteristics of micelles

**Fig. 2:**
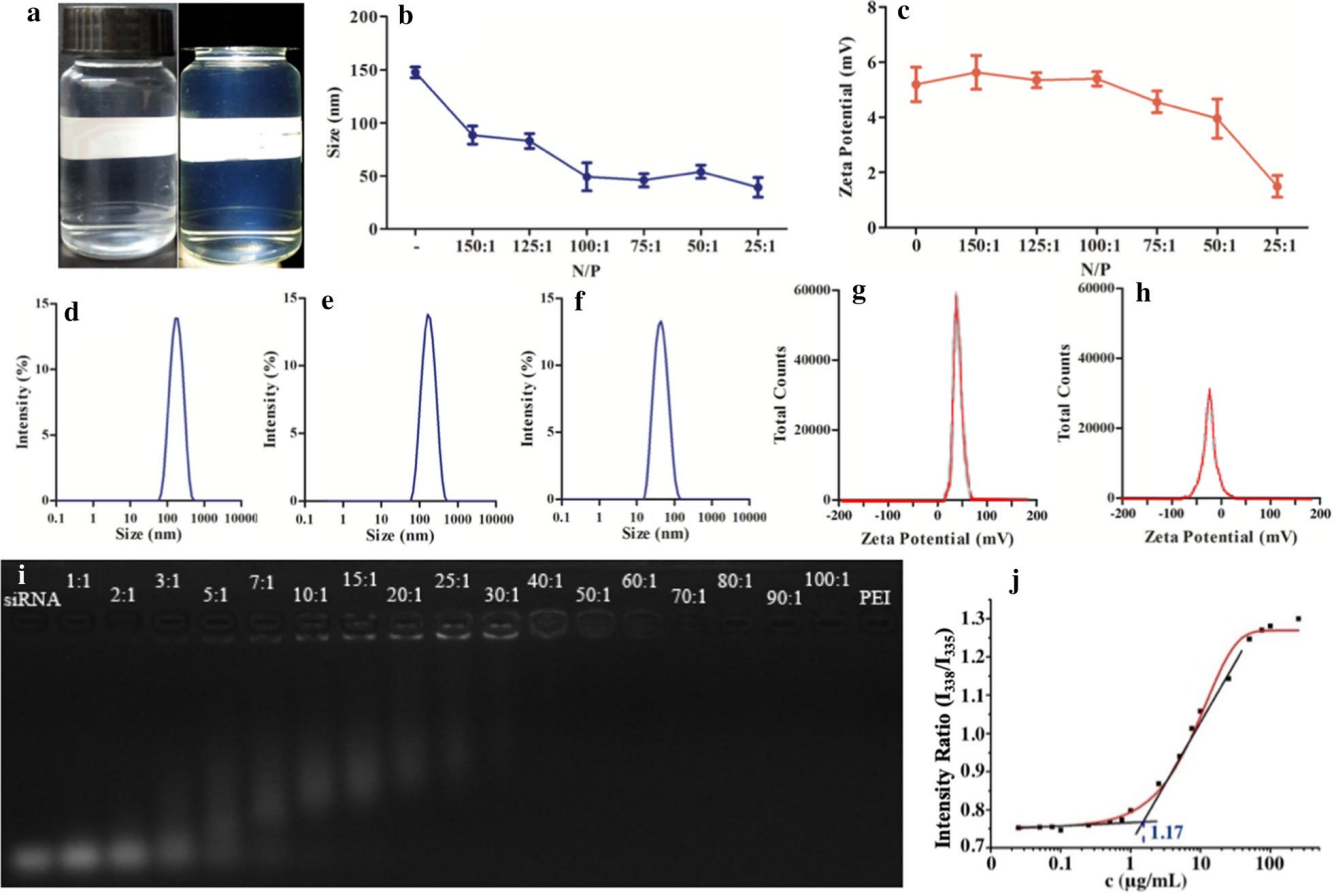
Characteristics of micelles. **a** Photograph of PEO-PCL micelles under natural light or under a beam of light (showing the Tyndall effect). **b** Size distribution of A-PEO-P(CL-SP) micelles loaded with siRNA at different N/P. **c** Zeta potential distribution of A-PEO-P(CL-SP) micelles loaded with siRNA at different N/P. **d**–**f** Size distribution of A-PEO-PCL blank micelles, A-PEO-P(CL-SP) blank micelles, and A-PEO-P(CL-SP) micelles loaded with siRNA. **g**–**h** Zeta potential distribution of A-PEO-P(CL-SP) blank micelles and A-PEO-P(CL-SP) micelles loaded with siRNA. **i** Agarose gel electrophoresis of A-PEO-P(CL-SP) loaded with siRNA at different N/P. **j** CMC profile of co-delivery micelles

Physical properties of polymer micelles: The obtained micelle solution was a colourless and transparent colloidal solution (Fig. 2a). The solution was transparent under natural illumination, and the Tyndall effect was obvious when a beam of laser light was applied for irradiation. The particle size of the A-PEO-PCL micelle loaded with DTX was 152.8 nm, and the PDI was 0.178. The size of the SP-modified micelles was 149 nm. After A-PEO-P(CL-SP) was mixed with anti-NS siRNA at different N/P ratios, the size substantially decreased from 149 nm to 42 nm (Fig. 2b, d, e, f). A-PEO-PCL had a zeta potential of − 12.8 mV, which increased to + 15 mV after modification with SP. The zeta potential of micelles with anti-NS siRNA at different N/P ratios decreased from 6.3 to 1.4 mV with the increase in siRNA (Fig. 2c, g, h).

The results of agarose gel electrophoresis are shown in Fig. 2i. With the increase in A-PEO-P(CL-SP), the bands became faded, and the location approached closer to the sample-loading holes. When the N/P ratio reached 25:1, the bands disappeared, similar to PEI as a positive control. The CMC was calculated as 1.17 µg/mL (Fig. 2j).

**Fig. 3:**
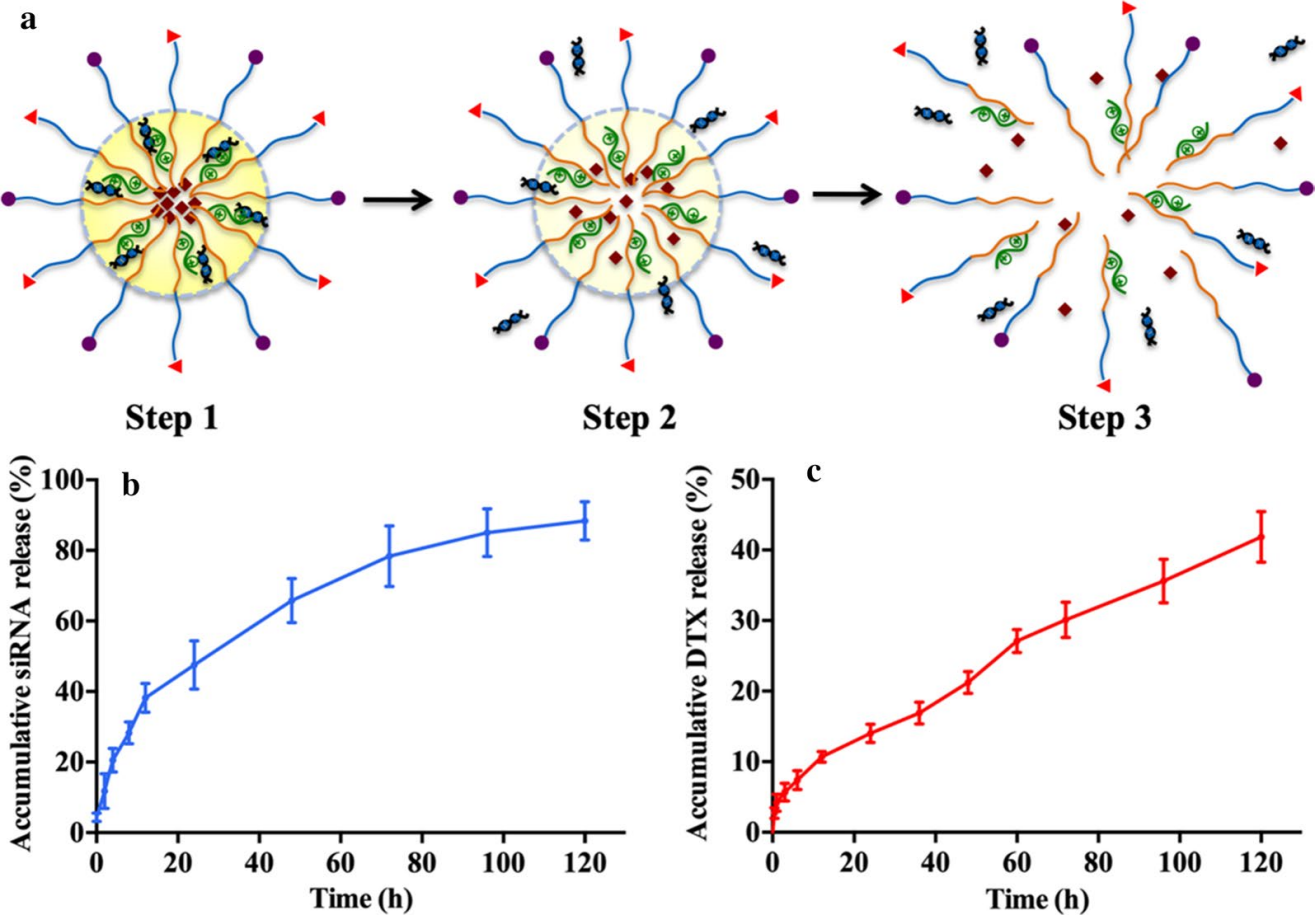
**a** Schematic processes of drug release in co-delivery micelles. **b** Anti-NS siRNA release curve in co-delivery micelles. **c** DTX release curve in co-delivery micelles

The drug loading rate of the micelles was 7.62%, and the encapsulation efficiency was 20.27%. The release of anti-NS siRNA was quick and stable. We found that 20.50% and 47.56% siRNA was released after 4 h and 24 h, respectively. After 72 h, the total cumulative siRNA release was 78.35%. However, the release of DTX was relatively slow, and the burst effect was not significant. A total of 12.5%, 19.50% and 28.02% DTX was released after 24 h, 48 h and 72 h, respectively. After 5 days, the total accumulative DTX release was only 41.84% (Fig. 3).

### In vitro experiment

**Fig. 4:**
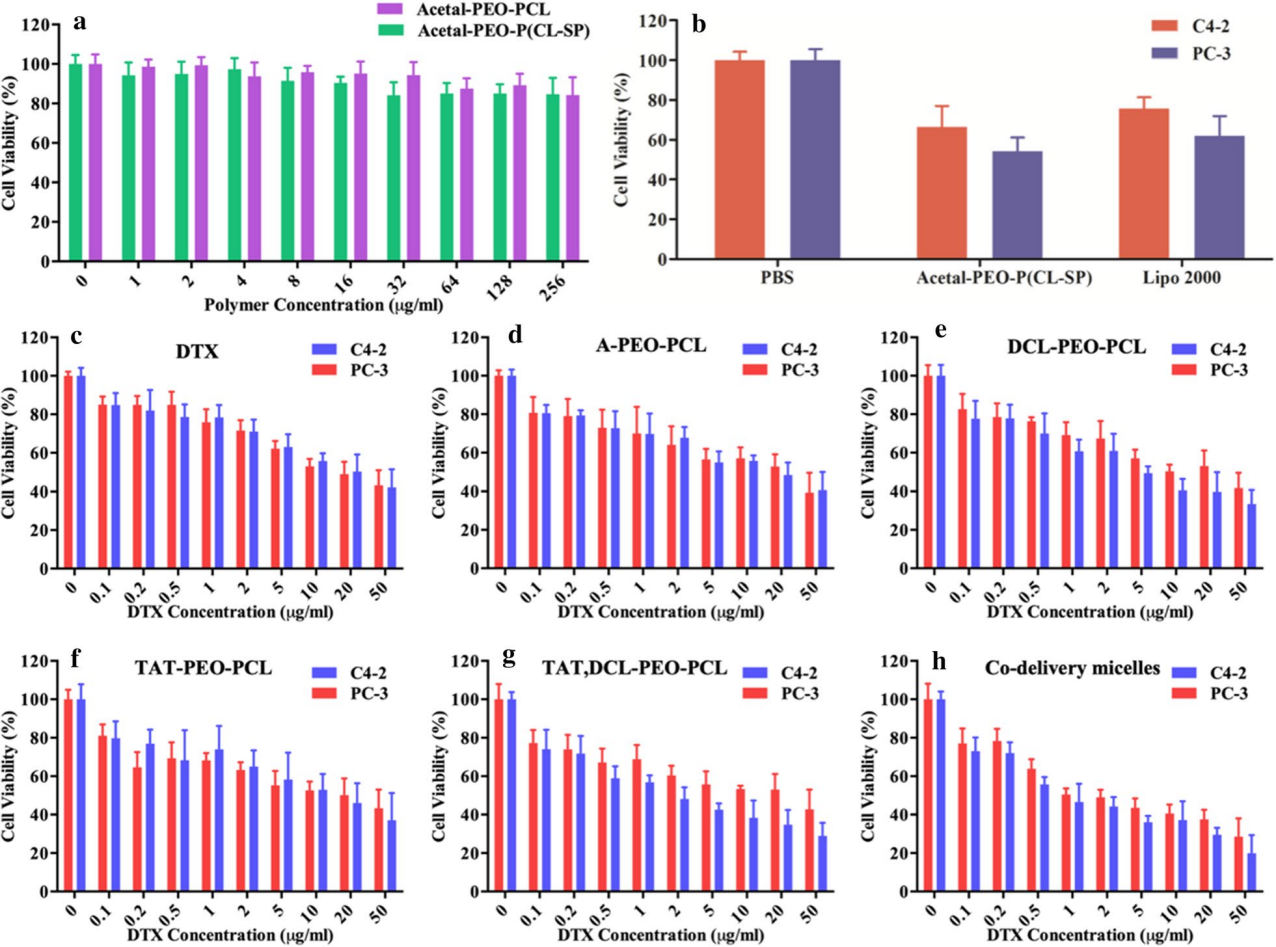
Cell viability after incubation with different micelles. **a** Toxicity of PEO-PCL and A-PEO-P(CL-SP) using the 3T3 cell line. **b**–**h** Cell viability after incubation with A-PEO-P(CL-SP) loaded with anti-NS siRNA (**b**), DTX (**c**), A-PEO-PCL micelles (**d**), DCL-PEO-PCL micelles (**e**), TAT-PEO-PCL micelles (**f**), DCL, TAT-PEO-PCL micelles (**g**), and co-delivery micelles (**h**) on C4-2 and PC-3 cells

Toxicity tests of the synthesized polymers showed that PEO-PCL was not significantly toxic to cells, regardless of SP modification. Even when the concentration increased to 256 µg/mL, the cell survival rate was greater than 84% in both groups (Fig. 4a). When micelles were used in gene transfer, the toxic effect was similar to that of the lipofectamine 2000 group after the same dose of anti-NS siRNA transferred (Fig. 4b). No significance was observed between the two groups in PC-3 (P = 0.197) and C4-2 (P = 0.116) cell lines.

To evaluate the effect of micelles with DTX, we first examined the toxicity of different concentrations of DTX on both cell lines (Fig. 4c–h). With the increase in DTX concentration, the viability of C4-2 and PC-3 cells decreased gradually to 42% and 43%, respectively. After entrapment in A-PEO-PCL micelles, a similar cell activity curve was obtained. However, when DCL was modified, the two curves separated, and the viability of C4-2 cells decreased (33.49%) to below that of the PC-3 cells (41.62%). After replacing DCL with TAT, the difference between the two lines disappeared, and the difference reappeared after mixing DCL-PEO-PCL with TAT-PEO-PCL to form micelles. Finally, targeted micelles with dual drugs resulted in the highest kill rate of C4-2 cells, and the survival rate dropped to 19.97% (28.57% compared with PC-3 cells).

**Fig. 5:**
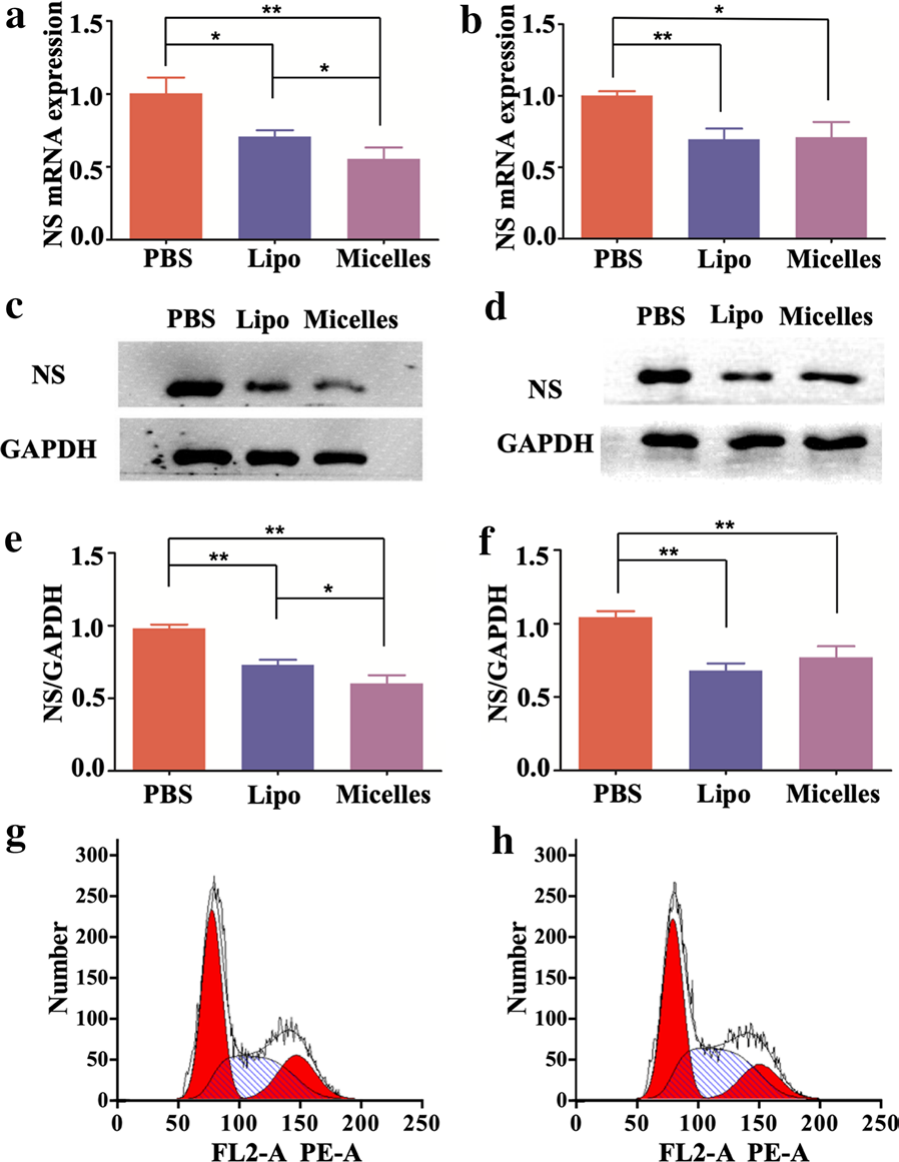
Expression of NS mRNA and protein. **a**, **b** qRT-PCR shows the expression of NS mRNA after micelle use in the C4-2 and PC-3 cell lines. **c**–**f** Western blot shows the expression of NS protein after micelle use in the C4-2 and PC-3 cell lines. **g**, **h** Flow cytometry shows the cell cycles before and after incubation with co-delivery of targeted micelles

The PCR results confirmed that co-delivery micelles significantly silenced the expression of the NS gene in C4-2 (P = 0.004) and PC-3 cell lines (P = 0.011) and achieved an effect similar to that of Lipo2000 (Fig. 5a, b). The C4-2 cells had much lower NS expression after incubation with co-delivery micelles (P = 0.043). No significant difference was noted in the PC-3 group (P = 0.853). The expression of NS protein was consistent with that of qPCR. Co-delivery micelles downregulated the NS protein in C4-2 (P = 0.001) and PC-3 cells (P = 0.005), and a significant difference was found in only the C4-2 group (0.032) (Fig. 5c–f).

Flow cytometry showed that the proportion of G0/G1 phase cells decreased from 45.40–43.23% in C4-2 cells treated with co-delivery targeted micelles. The proportion of G2/M phase cells decreased from 19.45–15.54%, and the proportion of S phase cells increased from 35.15–41.24% (Fig. 5g, h). There were significant differences in the proportions of the three different cell cycle stages (P < 0.05%).

### In vivo experiment

**Fig. 6:**
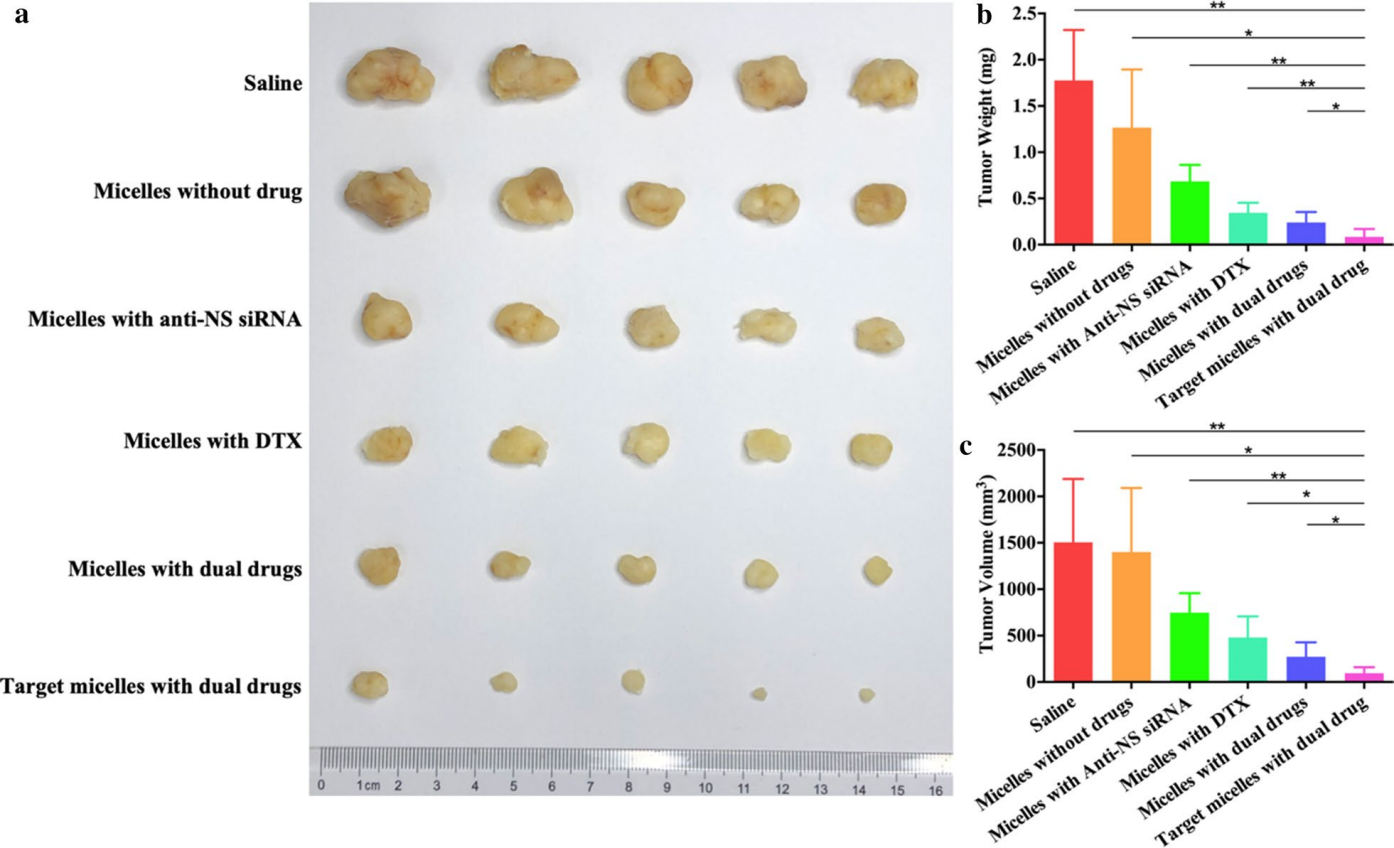
**a** Photograph of tumour tissues after 4-week treatment. **b** Tumour weights of each group. **c** Tumour volumes of each group. *p < 0.05 and **p < 0.01

A subcutaneous tumour model was established and treated with five types of micelles for 28 days. Among these treatments, the average tumour weight and volume after injection with drug-free micelles were similar to those of the saline group (P = 0.207, P = 0.818). After injection of micelles with anti-NS siRNA, micelles with DTX, or micelles with dual drugs, the tumour weight and volume were smaller than the former groups. However, mice in the targeted micelles with dual drugs group exhibited the smallest tumour weight and volume, and a significant difference was found compared to the other groups (P = 0.002, P = 0.013, P < 0.001, P = 0.003, P = 0.04) (Fig. 6).

**Fig. 7:**
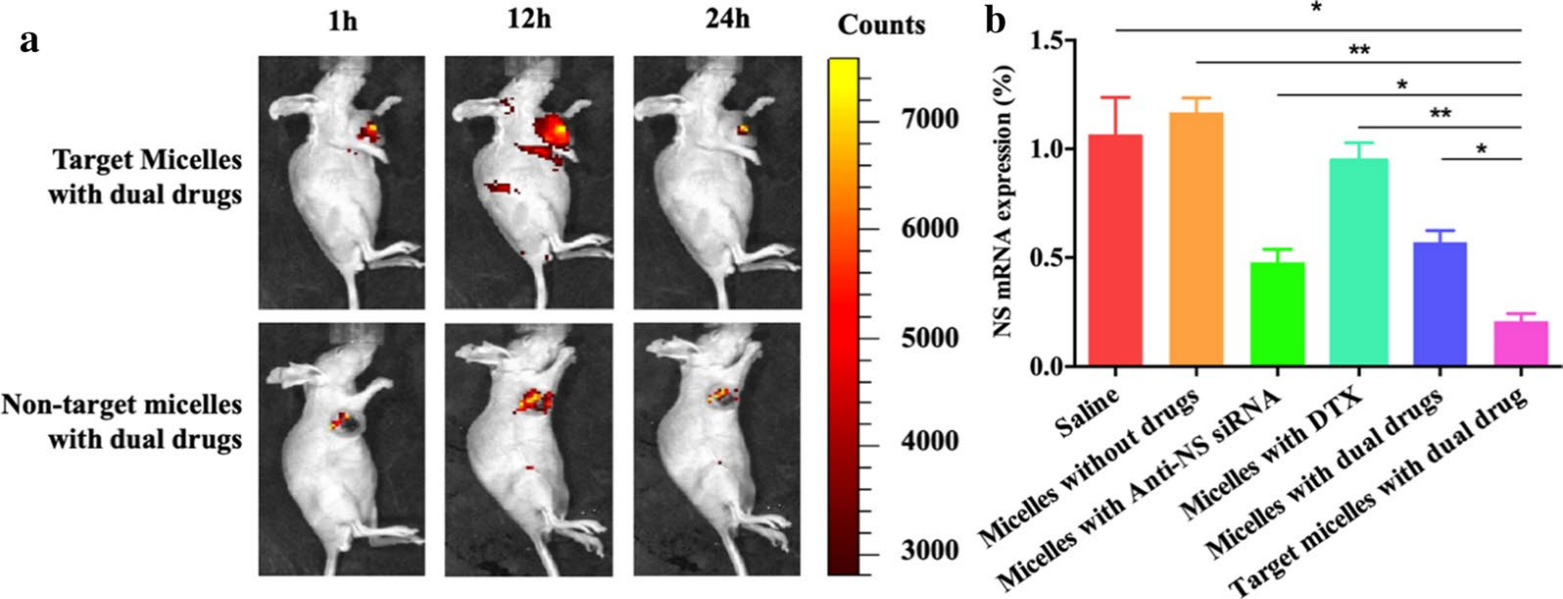
**a** In vivo imaging of tumour-burdened nude mice treated with co-delivery micelles at 1 h, 12 h and 24 h. **b** qRT-PCR quantification of NS mRNA expressed in tumour tissues. *p < 0.05 and **p < 0.01

To investigate the effect of the DCL ligand on the distribution of micelles *in vivo*, the IVIS results showed that the targeted micelles had stronger fluorescence intensity in the tumour area than micelles without DCL (Fig. 7a). This difference appeared 1 h after injection and became more significant after 12 h.

The PCR results of the tumour tissue showed that the expression of NS mRNA in the RNA-containing groups was significantly lower than that in the RNA-free groups (including saline, micelles without drugs, and micelles with DTX). The targeting group had the lowest NS mRNA expression compared with micelles in the anti-NS siRNA group (P = 0.046) and the micelles with dual drugs group (P = 0.016).

**Fig. 8:**
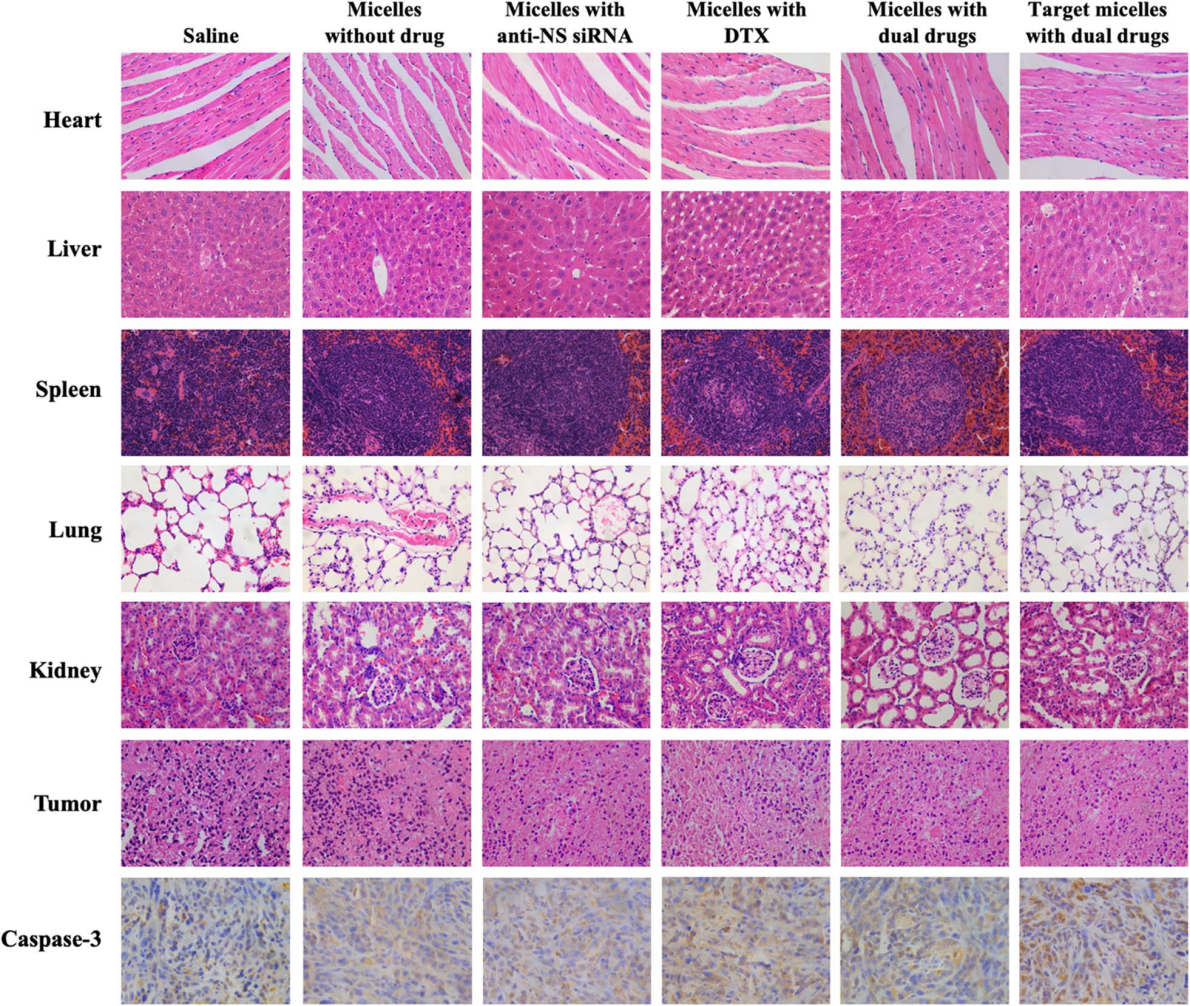
HE stains of heart, liver, spleen, lung, kidney and tumour tissue after treatment with saline, blank micelles, DTX-loaded micelles, anti-NS siRNA-loaded micelles, non-targeted micelles, and co-delivery targeted micelles. Immunohistochemistry of caspase-3 in tumour tissues (×200)

HE stains of the organs are shown in Fig. 8. No remarkable abnormalities were observed in the heart, liver, spleen, lung, kidney or tumours of each group. However, certain differences were noted in caspase-3 in the tumours. The introduction of DTX affected the apoptosis process of tumour cells.

Immunohistochemical staining showed the progress of tumour cell apoptosis in each group. Approximately 15.02% and 16.51% of cells were marked with caspase-3 in the negative group and no-drug group, and these values increased to 26.47% and 29.11% when anti-NS siRNA and DTX were introduced, respectively. Micelles with dual drugs showed a 35.25% positive rate, but the targeted micelles with dual drugs had the highest positive rate at 45.43%.

## Discussion

The present study designed, prepared and characterized a highly effective and novel targeted dual-drug delivery system. Specifically, gene therapy and chemotherapy were synchronously applied and achieved accurate and efficient treatment of CRPC in the G1/S and G2/M mitotic cycle.

The drugs clinically used in CRPC treatment are generally multi-disciplinary comprehensive treatments, including chemotherapy drugs, new second-line endocrine therapy, radionuclide and biological therapy. However, due to the limited scope of these treatments and higher costs, the number of patients who benefit is limited. Many elderly patients cannot tolerate adverse reactions, which greatly reduce the patient’s quality of life and survival benefit. An appropriate delivery system could effectively increase the drug delivery capacity and enable additional functions via chemical modification. Therefore, a better delivery vehicle that could reduce the side effects of chemotherapy drugs and introduce the latest gene therapy is necessary.

PEO-*b*-PCL is a widely used biological material, and its application forms include composite coatings [24], liposomes [25], electrospinning [26], mesoporous carbon [8], nanocapsules [27] and micelles [9]. The synthesis of PEO-PCL was also matured via a ring-opening polymerization of ε-caprolactone initiated by a PEO-bound lysine macroinitiator. Adjusting the ratio of PEO and PCL segments effectively changes the properties of the final product. After consulting the relevant research and conducting many pre-experiments, we selected a PEO:PCL ratio of 2:1 to supply the degree of freedom in space for the formation of a micelle structure. GPC was used to calculate the molecular weight and distribution of the polymers, and ^1^HNMR and FT-IR were performed for the structural characterization. Based on the high molecular weights of the PEO and PCL groups, the modification of other small molecules did not cause structural failure. An ideal CMC value ensured that the micelles maintained their structures and avoided destruction of micellar morphology when diluted in the blood after injection in vivo.

Modification of polymers supplies special biological functions. SP-modified micelles have positive charges and give micelles the ability to effectively carry genetic drugs, and RNA binding was fully combined at the N/P ratio of 25:1. N/P means the ratio of nitrogen to phosphorus. Nitrogen reflects the content of amino groups, which may be used to measure the content of polymers. Phosphorus reflects the content of phosphate groups, which may be used to measure the content of RNA. Therefore, N/P may be used to compare the approximate mixing ratio of polymer and RNA. The particle size, zeta potential and agarose gel electrophoresis results found that all of the parameters changed with alterations of the N/P ratio, and 75:1 was selected as the best N/P ratio for the efficient integration of genetic drugs and avoidance of RNA fugitives due to insufficient binding force. Agarose gel electrophoresis also demonstrated the best combination when the N/P reached 25:1 because the micelles entirely protected the siRNA. The appropriate particle size helps the micelles have selective high permeability and retention to pass through the tumour tissue, which has abundant blood vessels, wide vascular wall space, poor structural integrity and lack of lymphatic reflux.

The drug loading rate is generally lower in micelles due to the limitation of the micellar structure. We prepared micelles via dialysis, which was a relatively easy procedure. Especially for modified polymers, the molecular weight is generally relatively large, which also leads to a relatively low drug loading rate. Other researchers reported similar values of a 10% drug loading rate [28, 29], with certain reports reaching as high as 20% [30]. However, the ultrasonic method was used in preparation, and the molecular weight of the material was relatively small. The release of dual drugs within the specially designed structure could be programmable. The siRNA in the middle layer of the micelles was released earlier than the DTX in the core. Therefore, the anti-NS siRNA first suppressed the expression of NS and increased the effect of DTX. We found that nearly half of the siRNA was released after 24 h, while the release of DTX was 12.5%. We believe that this different release procedure is helpful for the synergistic interaction of genetic therapy and chemotherapy in vivo. The long-term DTX release process was also beneficial to drug accumulation in the target location because of the prolonged time of the effective DTX concentration.

PSMA is a transmembrane protein expressed on PCa epithelial cells [25, 26] with folate hydrolase I and glutamate carboxypeptidase II activity [31], and it directly correlated with PCa. PSMA expression is lower on normal prostate epithelial cell membranes, and it remains at a low level in the prostate gland cells of benign prostatic hyperplasia. PSMA is also expressed in the vascular epithelium of many other tumour tissues, but it is expressed at a low level in the vascular epithelium of normal tissues [32, 33]. These features make it a target for biomarkers, especially for the diagnosis and treatment of CRPC [34, 35]. The C4-2 cell line derived from CRPC expresses the PSMA receptor, but the PC-3 cell lacks expression. This difference allowed the two cell lines to be selected for comparison of the effects of targeted micelles. DCL is a small molecule ligand with the chemical name dicarboxypropyl-carbamoyl lysine (*N*-[*N*-[(S)-1,3-dicarboxypropyl]carbamoyl]-(S)-lysine), and the small molecule ligand is also known as S-5-amino-1-carboxy-3-pentyl-2-ureidoglutaric acid ((S)-2-(3-((S)-5)-amino-1-carboxypentyl) ureido) pentanedioic acid, SMLP) [36], which binds to PSMA via strong hydrogen bonds [17]. Many studies used DCL to link nanomaterials for the targeting of CRPC tissues. Hrkach et al. linked DCL to PEG-PLGA and validated it in cell and animal experiments. These researchers concluded that DCL molecules had CRPC targeting ability [37]. The DCL polypeptide was linked to the PEO end of the PEO-PCL micelle in this study to supply a targeting effect. The cellular study showed the targeting effect towards C4-2 cell lines, which was also demonstrated by the time-dependent biodistribution of micelles in vivo. This phenomenon demonstrated the targeting effect of the DCL ligand, which was also reported by Jin [36]. The connection of DCL cannot affect the macromolecular segment PEO-PCL because it is a tiny molecule compared to PEO-PCL.

The TAT peptide is an important group in micelles. The downward shift of the MTT curves indicated that the transmembrane effect of TAT assisted the micelles in entering the cell. The efficient cell penetration may be derived from energy-dependent endocytosis [38] and energy-independent cell penetration [39], which were reported by Jiménez-Mancilla and Peng [40, 41]. The TAT peptide strongly binds to the cell surface heparan sulphates and induces their aggregation via cross-linking [23].

Apoptosis is the manifestation of the therapeutic effect on tumour cells, and blockade of the mitotic cycle of cells is an effective mechanism for promoting apoptosis. Simple chemotherapy drugs block apoptosis from only one dimension, but targeted micelles with dual drugs greatly enhanced the effect. Depending on the downregulation of the NS gene and protein expression, this treatment had a synergistic effect in the G1/S and G2/M phases. Therefore, the expression level of caspase-3 was elevated after the combination of dual drugs. Under the action of the target and transmembrane peptide, the therapeutic effect was further improved, and the advantages of micelle modification were fully utilized, which significantly improved the treatment of CRPC.

This work has several limitations. (1) The system has many components and is relatively complicated to synthesize. A detailed study of the effect of the proportion of each component on the system might be required to improve the function of micelles. (2) The drug-carrying performance of micelles was not strong, and it was difficult to maintain an effective drug concentration in vivo over a long period of time. This observation is related to the structure of the micelle itself, and other structures, such as the nanoparticles, might be potential architectural forms.

## Conclusions

A new PEO-PCL-based drug delivery system was designed and modified as a carrier for DTX and anti-NS siRNA in the treatment of CRPC. Different functions were achieved by each single group. Spermine groups offer electro-positivity, and the DCL peptide helps the targeting of prostate cancer cells. The TAT peptide intensifies cell penetration. Due to the synergistic effect of the genetic and chemical components, the entire system effectively enhanced cell apoptosis by inhibiting the G1/S and G2/M mitotic cycle. The oriented DCL peptide guided the system to the PSMA-positive CRPC tissue where androgenic drugs were insensitively deployed. Overall, this targeted dual-drug delivery system offers a viable option for the treatment of terminal cancer and presents an idea for the future design of dual-drug delivery systems.

## Materials and methods

### Materials

SP was purchased from J&K Scientific Ltd. DCL was purchased from Chinese Peptide (Hangzhou, China). TAT peptide was purchased from GL Biochem Corporation Ltd. (Shanghai, China). Anti-NS siRNA was purchased from GenePharma (Shanghai, China). Acetal-PEO (MW = 8655 g/mol) was obtained from the Tianjin Chemical Reagent No. 6 factory (Tianjin, China). The SDS-PAGE, MTT kits, formaldehyde solution (4%), and HE staining solution were obtained from Beyotime (Shanghai, China) and Solarbio (Beijing, China). Other chemicals and reagents were purchased from Sigma-Aldrich (USA). RPMI 1640 cell culture medium, foetal bovine serum (FBS) and other cell culture reagents were obtained from Gibco (Shanghai, China). Biological antibodies were purchased from Abcam plc (UK) and Proteintech (USA).

### Preparation of polymers

**Scheme 2:**
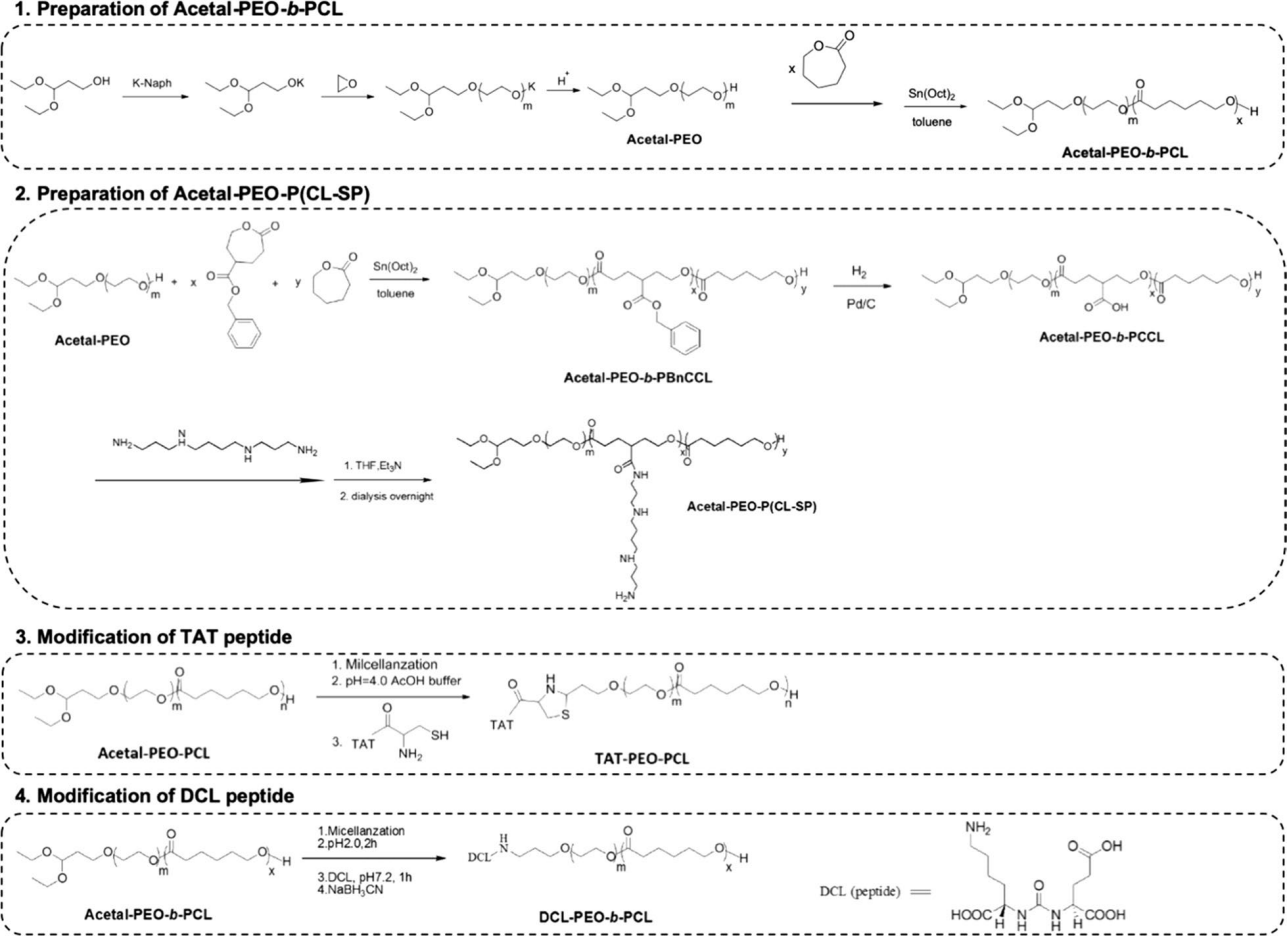
Chemical equations of the preparation processes for PEO-PCL, DCL ligand and TAT peptide

The chemical equations for the preparation and modification processes are listed in Scheme 2.

Preparation of A-PEO-PCL: A-PEO-PCL was synthesized by open-ring polymerization. One gram of acetal-PEO (MW = 8655 g/mol) was dissolved in anhydrous toluene as a macroinitiator. A certain amount of caprolactone was added to initiate the reaction, and stannous octoate was used as a catalyst. The system was constantly stirred at 120℃ for 24 h, and the reaction was subsequently stopped and cooled. The product was precipitated in methanol and filtered to obtain A-PEO-PCL powder.

Synthesis of A-PEO-P(CL-SP): Two grams of acetal-PEO was dissolved in 5 mL toluene as a macroinitiator, and 0.25 g caprolactone and 0.23 g BnCCL were added to the system. Under the same reaction conditions previously described, stannous octoate was used as a catalyst. After 24 h, the obtained product was purified in cold methanol to obtain pure acetal-PEO-PBnCCL. The product was dissolved in THF, and H_2_ was introduced into the system. Bn was removed with the catalysing function of Pd/C. After filtration and purification, A-PEO-PCCL was obtained and modified with spermine via the coupling of amino and carboxyl groups. Briefly, 200 mg A-PEO-PCCL was dissolved in THF and EDC. HCl was added, and the solution was stirred for 30 min. NHS was added, and the solution was stirred for 2 h. Excessive SP was added, and the solution was stirred overnight. THF and unreacted SP were removed via dialysis. The system was freeze-dried to obtain A-PEO-P(CL-SP).

Modification of the TAT peptide: A certain amount of A-PEO-PCL powder was weighed to prepare a micelle solution with a concentration of 2.0 mg/mL, and 5% HCl was used to adjust the pH to 2.0. The mixture was stirred for 2 h under ambient temperature to remove the aldehyde. Subsequently, 20% NaOH was applied to adjust the pH to 4.0. Excessive TAT liquid was added and stirred overnight at room temperature. The product was dialysed with a dialysis bag (MWCO = 10 kD) to remove the non-reactive TAT polypeptide and buffer salt. After filtering, ultra-filtering and freeze-drying, TAT-PEO-PCL was obtained.

Modification of the DCL target: Similar to modification of the TAT ligand, after removing the aldehyde of A-PEO-PCL, excessive DCL liquid was added, and the solution was stirred overnight at room temperature. Sodium borohydride was added as a reducing agent, and the solution was stirred for 2 h. DCl-PEO-PCL was obtained after dialysing, filtering and freeze-drying.

### Characteristics of polymers

^1^H nuclear magnetic resonance (^1^HNMR) measurement: Ten milligrams of A-PEO-PCL, A-PEO-P(CL-SP), TAT-PEO-PCL, and DCL-PEO-PCL were separately and fully dissolved with 0.5 mL deuterium chloride in nuclear magnetic tubes. A Bruker 400 MHz NMR (Switzerland) instrument was applied to measure the ^1^HNMR spectra.

GPC measurement: Five milligrams each of A-PEO-PCL, A-PEO-P(CL-SP), TAT-PEO-PCL, and DCL-PEO-PCL was dissolved in chromatographic grade THF and filtered after ultrasonic degassing. Polystyrene was selected as the standard sample. After injection into a gel chromatography column (Waters, USA), the elution time was measured, and the molecular weight was calculated.

Fourier transform infrared spectroscopy (FT-IR) measurement: Dry potassium bromide and a small amount of dried sample powder were ground into a powder. The two compounds were pressed into tablets until evenly mixed. Infrared spectroscopy was performed by placing the tablet in an infrared spectrometer (Bruker, Germany).

### Self-assembly and characteristics of micelles

Self-assembly of micelles by dialysis: Twenty milligrams of PEO-P(CL-SP), 10 mg DCL-PEO-PCL, 10 mg TAT-PEO-PCL, and 3 mg DTX were dissolved in 4 mL tetrahydrofuran in a spherical bottle at room temperature overnight. To this solution, 0.1% DEPC and 30 µg anti-NS siRNA were added under 1000 rpm stirring. The solution was slowly pumped into 60 mL of water at a speed of 15 mL/h and a temperature of 35 °C. The liquid was dialysed with a dialysis bag (molecular weight of 10 kD). The micelle solution was filtered through a 0.45-µm cellulose filter and ultra-filtered with a molecular weight cut-off of 10 kD.

Size and zeta potential: The micelle solution was prepared, and all samples were adjusted to 0.5 mg/mL. The particle size and zeta potential were measured 3 times using a Malvern Nano ZetaSizer.

Agarose gel electrophoresis: Anti-NS siRNA (0.2 µg) was dissolved in enzyme-free PBS buffer and added to enzyme-free EP tubes. Acetal-PEO-P(CL-SP) was dissolved and mixed with siRNA to reach a gradient N/P ratio from 1:1 to 100:1. A blank negative control (siRNA only) and PEI positive control (100 µg PEI2500 and 0.2 µg siRNA) were also prepared. Enzyme-free PBS was added to reach a 10 µL volume, and the system was fully mixed, vortexed for 1 min and incubated for 30 min. Samples were separately mixed with loading buffer and loaded onto a 1.5% (w/v) agarose gel pre-stained with Gel Red. The electrophoresis apparatus was set at 100 V for 30 min, and the bands were visualized.

Critical micelle concentration: Pyrene acetone solution (180 µL, 1 × 10^− 5^ mol/L) was placed in brown glass bottles, and the acetone was completely evaporated with avoidance of light and ventilation, at which time the pyrene content in each bottle was 1.8 × 10^− 6^ mol. Micelle solutions with different concentrations were separately added to the bottles to ensure that the pyrene concentration was 6.0 × 10^− 7^ mol/L. The bottles were shaken at 37 °C while avoiding light to reach equilibrium. The excitation spectra of each sample were measured at an emission wavelength of 393 nm. The critical micelle concentration was calculated according to the result of I_338_/I_335_.

Drug loading and release: The targeted micelle solution with dual drugs was prepared, lyophilized and accurately weighed. The concentration of DTX was determined using a high-performance liquid chromatograph (HPLC) (Waters, USA). DTX standards with different concentrations were dissolved in acetonitrile, degassed by ultrasound and filtered with 0.22-µm membrane. The detection wavelength of HPLC was set at 230 nm, the flow rate was 1 mL/min, the column temperature was 30 °C, and the flow phase ratio was 90:10 (acetonitrile:water). After the baseline was stable, the detection was started with the injection volume of 10 µL each time. The peak area at 6–10 min was determined. The standard curve of DTX in acetonitrile dissolution was drawn from the detection of all standard samples. The prepared micelles were freeze-dried and dissolved in acetonitrile, and the peak area was calculated from the HPLC data. The solubility of DTX was calculated from the standard curve equation. The formulas for calculating the drug loading rate and encapsulation efficiency are listed as follows:

$${\text{Drug loading rate}} = {\text{DTX concentration measured by HPLC}} / \text{total concentration of drug-loaded micelles}*100\%$$

$$\text{Encapsulation efficiency} = \text{DTX concentration measured by HPLC/total amount of DTX when prepared}*100\%$$

The DTX release was tested using the dialysis method. The micelle powder containing 50 µg DTX was accurately weighed and dissolved in 300 µL PBS solution. The drug-loaded micelle solution was put into a dialysis bag (molecular weight of 10 kD) and immersed in 40 mL PBS solution containing 0.5% (w/v) Tween 80 at pH 7.4. The total amount of DTX in the dialysis solution was determined by HPLC at different times, and the total amount and rate of released DTX was calculated.

To confirm the inclusion of siRNA, co-delivery micelles were prepared with anti-NS siRNA labelled with FAM. The fluorescence intensity was tested by flow cytometry (BD LSRFortessa™, USA). The excitation and emission wavelengths were 480 nm and 520 nm, respectively. UV measurements were used to clarify siRNA release after dissolution in PBS solution.

### In vitro experiments

The PC-3 and C4-2 cell lines were cultured in RPMI-1640 medium supplemented with 10% FBS. The NIH 3T3 cell line was cultured in DMEM supplemented with 10% FBS at 37℃ in a 5% CO_2_ atmosphere.

MTT: The 3T3, PC-3 and C4-2 cells were seeded in 96-well plates with 80 µL medium and 6000 cells per well. Micelles dissolved in PBS at different concentrations were added to each well. After 48 h, the MTT solution was added and incubated for another 4 h. The liquid in each well was discarded, and 150 µL DMSO was added. The plate was placed on a low-speed shaker for 10 min. The OD value was recorded using a microplate reader at 570 nm wavelength to calculate the cell viability based on the OD value of PBS.

PCR: The level of NS mRNA was analysed by quantitative real-time polymerase chain reaction (qRT-PCR). Total RNA was extracted by TRIzol reagent (Invitrogen, Carlsbad, CA, USA). qRT-PCR was performed using SuperScript® one-step qRT-PCR according to the manufacturer’s protocol. All data were analysed using GAPDH as an internal standard. The NS primer sequences were 5′-ATGAAAAGGCCTAAGTTAAAGAAAGC-3′ (forward) and 5′-ATGAAAAGGCCTAAGTTAAAGAAAGC-3′ (reverse). GAPDH was used as an internal standard, and the primer sequences were 5′-GGTGGACCTGACCTGCCGTCTAGA-3′ (forward) and 5′-TTACTCCTTGGAGGCCATGTGGG-3’ (reverse).

Western blotting: PC-3 or C4-2 cells were seeded in 6-well plates with 3 mL medium and 2*10^5^ cells per well and treated with micelle solution for 48 h. RIPA lysis buffer and PMSF (Thermo Fisher Scientific, USA) were used to extract the cellular protein. SDS-PAGE and a poly-vinylidene fluoride membrane were used to separate the denatured protein. Non-fat milk (5%) was applied for blocking, and NS antibodies (Proteintech) were used to bond the NS protein overnight at 4 °C. The membranes were incubated with secondary horseradish peroxidase-conjugated antibodies for 1 h. The bands were visualized by enhanced chemiluminescence.

Cell cycle test: C4-2 cells were seeded in 6-well plates with 3 mL medium and 2*10^5^ cells per well and treated with micelle solution for 72 h. The cells were centrifuged and collected and fixed with 70% ethanol solution. After washing, 500 µL PBS solution containing 50 µg/mL ethidium bromide (PI), 100 µg/mL RNase A and 0.2% Triton X-100 was added. The cells were incubated in dark at 4℃ for 30 min. The cells were counted by flow cytometry.

### In vivo experiments

Six-week-old male BALB/c nude mice were purchased from the National Institute of Food and Drug Control (Beijing) and fed under specific pathogen-free (SPF) conditions at the Institute of Radiation Medicine Chinese Academy of Medical Sciences (Tianjin). The cell suspension with 10 µL medium and 2*10^7^ C4-2 cells were injected into the right flank to build the subcutaneously implanted tumour model in nude mice. After 2 weeks, all nude mice bearing tumours were randomly divided into 6 groups. Saline, micelles without drugs, micelles with anti-NS siRNA, micelles with DTX, micelles with dual drugs, and targeted micelles with dual drugs were injected via the tail vein every 4 days. The DTX dose was 0.5 mg/kg, and the anti-NS siRNA dose was 0.2 mg/kg. After 5 injections, the body weight and tumour size (V = π/6((Length + Width)/2)^3^) were recorded every 5 days.

An in vivo imaging system (IVIS) was used to show the biodistribution of micelles. Cy5.5-linked micelles and cy5.5-linked targeted micelles were injected into nude mice via the tail vein. All images were captured at a wavelength of 650 nm at 1 h, 12 h and 24 h after injection.

All nude mice were euthanized 28 days after injections. The tumours were weighed, and organs, including hearts, liver, spleen, lungs, and kidneys, were removed for wax block embedding, sectioning and HE staining. Immunohistochemical staining was also performed using caspase-3.

### Statistics

For statistical analyses between two groups, Student’s t-test was used, and P-values < 0.05 was considered significant. Statistical analyses were performed using SPSS software (SPSS Inc., Chicago, USA).

## Data Availability

All data generated or analysed during this study are included in this article.

## Abbreviations

CRPC: Castration-resistant prostate cancer
PSMA: Prostate-specific membrane antigen
DTX: Docetaxel
Anti-NS siRNA: Anti-nucleostemin small interfering RNA
PEO: Polyethylene oxide
PCL: Polycaprolactone
SP: Spermine
DCL: *N*-[*N*-[(S)-1,3-dicarboxypropyl]carbamoyl]-(S)-lysine
TAT: HIV-1 TAT Protein Peptide
A-PEO: Acetal-*b*-PEO
A-PEO-PCL: Acetal-*b*-PEO-*b*-PCL
A-PEO-P(CL-SP): Acetal-*b*-PEO-*b*-P(CL-SP)
DCL-PEO-PCL: DCL-*b*-PEO-*b*-PCL
TAT-PEO-PCL: TAT-*b*-PEO-*b*-PCL
C4-2: PCa cells that express PSMA receptor
PC-3: PCa cells without PSMA receptor expression

## References

[1] Siegel RL, Miller KD, Jemal A (2019). Cancer statistics, 2019. Cancer J Clin.

[2] Cornford P, Bellmunt J, Bolla M, Briers E, De Santis M, Gross T, Henry AM, Joniau S, Lam TB, Mason MD (2017). EAU-ESTRO-SIOG guidelines on prostate cancer. Part II: treatment of relapsing, metastatic, and Castration-resistant prostate cancer. Eur Urol.

[3] Sweeney CJ, Chen YH, Carducci M, Liu G, Jarrard DF, Eisenberger M, Wong YN, Hahn N, Kohli M, Cooney MM (2015). Chemohormonal therapy in metastatic hormone-sensitive prostate cancer. N Engl J Med.

[4] Baker J, Ajani J, Scotte F, Winther D, Martin M, Aapro MS, von Minckwitz G (2009). Docetaxel-related side effects and their management. Eur J Oncol Nurs.

[5] Bouman-Wammes EW, van den Berg HP, de Munck L, Beeker A, Smorenburg CH, Vervenne WL, Coenen JLLM, Verheul HMW, Gerritsen WR (2018). Van den Eertwegh AJM: A randomised phase II trial of docetaxel versus docetaxel plus carboplatin in patients with castration-resistant prostate cancer who have progressed after response to prior docetaxel chemotherapy: The RECARDO trial. Eur J Cancer.

[6] Doane TL, Burda C (2012). The unique role of nanoparticles in nanomedicine: imaging, drug delivery and therapy. Chem Soc Rev.

[7] Figarol A, Gibot L, Golzio M, Lonetti B, Mingotaud AF, Rols MP (2018). A journey from the endothelium to the tumor tissue: distinct behavior between PEO-PCL micelles and polymersomes nanocarriers. Drug Deliv.

[8] Chu WC, Bastakoti BP, Kaneti YV, Li JG, Alamri HR, Alothman ZA, Yamauchi Y, Kuo SW (2017). Tailored design of bicontinuous gyroid mesoporous carbon and nitrogen-doped carbon from poly(ethylene oxide-b-caprolactone) diblock copolymers. Chemistry.

[9] Hisey B, Ragogna PJ, Gillies ER (2017). Phosphonium-functionalized polymer micelles with intrinsic antibacterial activity. Biomacromol.

[10] Liu RL, Zhang ZH, Xu Y (2010). Downregulation of nucleostemin causes G1 cell cycle arrest via a p53-independent pathway in prostate cancer PC-3 Cells. Urol Int.

[11] Liu RL, Zhang ZH, Zhao WM, Wang M, Qi SY, Li J, Zhang Y, Li SZ, Xu Y (2008). Expression of nucleostemin in prostate cancer and its effect on the proliferation of PC-3 cells. Chin Med J.

[12] Huang G, Meng L, Tsai RY (2015). p53 configures the G2/M arrest response of nucleostemin-deficient cells. Cell Death Discov.

[13] Ficker E, Taglialatela M, Wible, Henley BA, Brown CM (1994). Spermine and spermidine as gating molecules for inward rectifier K + channels. Science.

[14] Fakler B, BräNdle U, Glowatzki, Weidemann E, Zenner S, Ruppersberg HP (1995). Strong voltage-dependent inward rectification of inward rectifier K + channels is caused by intracellular spermine. Cell.

[15] Remy JS, Sirlin C, Vierling P, Behr JP (1994). Gene transfer with a series of lipophilic DNA-binding molecules. Bioconjug Chem.

[16] Shen Y, Wang B, Lu Y, Ouahab A, Li Q, Tu J (2011). A novel tumor-targeted delivery system with hydrophobized hyaluronic acid-spermine conjugates (HHSCs) for efficient receptor-mediated siRNA delivery. Int J Pharm.

[17] Sanna V, Pintus G, Bandiera P, Anedda R, Punzoni S, Sanna B, Migaleddu V, Uzzau S, Sechi M (2011). Development of polymeric microbubbles targeted to prostate-specific membrane antigen as prototype of novel ultrasound contrast agents. Mol Pharm.

[18] Sanna V, Singh CK, Jashari R, Adhami VM, Chamcheu JC, Rady I, Sechi M, Mukhtar H, Siddiqui IA (2017). Targeted nanoparticles encapsulating (-)-epigallocatechin-3-gallate for prostate cancer prevention and therapy. Sci Rep.

[19] Haberkorn U, Eder M, Kopka K, Babich JW, Eisenhut M (2016). New strategies in prostate cancer: prostate-specific membrane antigen (PSMA) ligands for diagnosis and therapy. Clin Cancer Res.

[20] Pantel K, Alix-Panabieres C (2012). The potential of circulating tumor cells as a liquid biopsy to guide therapy in prostate cancer. Cancer Discov.

[21] Sweat SD, Pacelli A, Murphy GP, Bostwick DG (1998). Prostate-specific membrane antigen expression is greatest in prostate adenocarcinoma and lymph node metastases. Urology.

[22] Hofman MS, Violet J, Hicks RJ, Ferdinandus J, Thang SP, Akhurst T, Iravani A, Kong G, Ravi Kumar A, Murphy DG (2018). [(177)Lu]-PSMA-617 radionuclide treatment in patients with metastatic castration-resistant prostate cancer (LuPSMA trial): a single-centre, single-arm, phase 2 study. Lancet Oncol.

[23] Ziegler A, Seelig J (2004). Interaction of the protein transduction domain of HIV-1 TAT with heparan sulfate: binding mechanism and thermodynamic parameters. Biophys J.

[24] Tian P, Xu D, Liu X (2016). Mussel-inspired functionalization of PEO/PCL composite coating on a biodegradable AZ31 magnesium alloy. Colloids Surf B Biointerfaces.

[25] Pippa N, Naziris N, Stellas D, Massala C, Zouliati K, Pispas S, Demetzos C, Forys A, Marcinkowski A, Trzebicka B (2019). PEO-b-PCL grafted niosomes: The cooperativilty of amphiphilic components and their properties in vitro and in vivo. Colloids Surf B Biointerfaces.

[26] Eskitoros-Togay SM, Bulbul YE, Tort S, Demirtas Korkmaz F, Acarturk F, Dilsiz N (2019). Fabrication of doxycycline-loaded electrospun PCL/PEO membranes for a potential drug delivery system. Int J Pharm.

[27] Maglio G, Nicodemi F, Conte C, Palumbo R, Tirino P, Panza E, Ianaro A, Ungaro F, Quaglia F (2011). Nanocapsules based on linear and Y-shaped 3-miktoarm star-block PEO-PCL copolymers as sustained delivery system for hydrophilic molecules. Biomacromol.

[28] Cheng Q, Du L, Meng L, Han S, Wei T, Wang X, Wu Y, Song X, Zhou J, Zheng S (2016). The Promising Nanocarrier for Doxorubicin and siRNA Co-delivery by PDMAEMA-based Amphiphilic Nanomicelles. ACS Appl Mater Interfaces.

[29] Xin X, Lin F, Wang Q, Yin L, Mahato RI (2019). ROS-responsive polymeric micelles for triggered simultaneous delivery of PLK1 inhibitor/miR-34a and effective synergistic therapy in pancreatic cancer. ACS Appl Mater Interfaces.

[30] Mao W, Mao D, Yang F, Ma D (2019). Transformative Supramolecular Vesicles Based on Acid-Degradable Acyclic Cucurbit[n]uril and a Prodrug for Promoted Tumoral-Cell Uptake. Chemistry.

[31] Maresca KP, Hillier SM, Femia FJ, Keith D, Barone C, Joyal JL, Zimmerman CN, Kozikowski AP, Barrett JA, Eckelman WC, Babich JW (2009). A series of halogenated heterodimeric inhibitors of prostate specific membrane antigen (PSMA) as radiolabeled probes for targeting prostate cancer. J Med Chem.

[32] Chang SS, O’Keefe DS, Bacich DJ, Reuter VE, Heston WD, Gaudin PB (1999). Prostate-specific membrane antigen is produced in tumor-associated neovasculature. Clin Cancer Res.

[33] Ghosh A, Heston WD (2004). Tumor target prostate specific membrane antigen (PSMA) and its regulation in prostate cancer. J Cell Biochem.

[34] Colombatti M, Grasso S, Porzia A, Fracasso G, Scupoli MT, Cingarlini S, Poffe O, Naim HY, Heine M, Tridente G (2009). The prostate specific membrane antigen regulates the expression of IL-6 and CCL5 in prostate tumour cells by activating the MAPK pathways. PLoS One.

[35] Wolf P, Freudenberg N, Buhler P, Alt K, Schultze-Seemann W, Wetterauer U, Elsasser-Beile U (2010). Three conformational antibodies specific for different PSMA epitopes are promising diagnostic and therapeutic tools for prostate cancer. Prostate.

[36] Jin J, Sui B, Gou J, Liu J, Tang X, Xu H, Zhang Y, Jin X (2014). PSMA ligand conjugated PCL-PEG polymeric micelles targeted to prostate cancer cells. PLoS ONE.

[37] Hrkach J, Von Hoff D, Mukkaram Ali M, Andrianova E, Auer J, Campbell T, De Witt D, Figa M, Figueiredo M, Horhota A (2012). Preclinical development and clinical translation of a PSMA-targeted docetaxel nanoparticle with a differentiated pharmacological profile. Sci Transl Med.

[38] Vives E (2003). Cellular uptake [correction of utake] of the Tat peptide: an endocytosis mechanism following ionic interactions. J Mol Recognit.

[39] Jarver P, Langel U (2004). The use of cell-penetrating peptides as a tool for gene regulation. Drug Discov Today.

[40] Jimenez-Mancilla N, Ferro-Flores G, Santos-Cuevas C, Ocampo-Garcia B, Luna-Gutierrez M, Azorin-Vega E, Isaac-Olive K, Camacho-Lopez M, Torres-Garcia E (2013). Multifunctional targeted therapy system based on (99 m) Tc/(177) Lu-labeled gold nanoparticles-Tat(49–57)-Lys(3) -bombesin internalized in nuclei of prostate cancer cells. J Labelled Comp Radiopharm.

[41] Peng LH, Niu J, Zhang CZ, Yu W, Wu JH, Shan YH, Wang XR, Shen YQ, Mao ZW, Liang WQ, Gao JQ (2014). TAT conjugated cationic noble metal nanoparticles for gene delivery to epidermal stem cells. Biomaterials.

